# Ammonia deposition in the neighbourhood of an intensive cattle feedlot in Victoria, Australia

**DOI:** 10.1038/srep32793

**Published:** 2016-09-07

**Authors:** Jianlin Shen, Deli Chen, Mei Bai, Jianlei Sun, Trevor Coates, Shu Kee Lam, Yong Li

**Affiliations:** 1 Key Laboratory of Agro-Ecological Processes in Subtropical Regions, Institute of Subtropical Agriculture, Chinese Academy of Sciences, Changsha 410125, China; 2 Faculty of Veterinary and Agricultural Sciences, The University of Melbourne, Victoria 3010, Australia

## Abstract

Intensive cattle feedlots are large emission sources of ammonia (NH_3_), but NH_3_ deposition to the landscape downwind of feedlots is not well understood. We conducted the first study in Australia to measure NH_3_ dry deposition within 1 km of a commercial beef cattle feedlot in Victoria. NH_3_ concentrations and deposition fluxes decreased exponentially with distance away from the feedlot. The mean NH_3_ concentrations decreased from 419 μg N m^−3^ at 50 m to 36 μg N m^−3^ at 1 km, while the mean NH_3_ dry deposition fluxes decreased from 2.38 μg N m^−2^ s^−1^ at 50 m to 0.20 μg N m^−2^ s^−1^ at 1 km downwind from the feedlot. These results extrapolate to NH_3_ deposition of 53.9 tonne N yr^−1^ in the area within 1 km from the feedlot, or 67.5 kg N ha^−1^ yr^−1^ as an area-weighted mean, accounting for 8.1% of the annual NH_3_-N emissions from the feedlot. Thus NH_3_ deposition around feedlots is a significant nitrogen input for surrounding ecosystems. Researches need be conducted to evaluate the impacts of NH_3_ deposition on the surrounding natural or semi-naturals ecosystems and to reduce N fertilizer application rate for the surrounding crops by considering nitrogen input from NH_3_ deposition.

Ammonia (NH_3_) is the primary alkaline gas in the air. Once emitted into the atmosphere, part of it can be removed through dry deposition over the areas near the emission source^1^,^2^. The remaining NH_3_ can react with acidic gases such as H_2_SO_4_ and HNO_3_ to form secondary aerosols, including (NH_4_)_2_SO_4_, NH_4_HSO_4_ and NH_4_NO_3_, which are the major contributors to fine particulate matter^3^,^4^. These secondary aerosols can be transported long distances and eventually removed from the air by dry and wet deposition^2^,^5^. The deposition of NH_3_ and its secondary aerosols can result in increased nitrogen (N) input to the natural or semi-natural ecosystems and subsequently eutrophication^6^, soil acidification^7^ and loss of species diversity^8^.

Atmospheric NH_3_ is emitted mainly from anthropogenic sources^9^, with animal production facilities as the major source of atmospheric NH_3_ in many developed countries^1^,^10^,^11^,^12^. A few studies have reported on NH_3_ deposition near hotspots such as animal farms. For example, Fowler *et al*.^13^ monitored the NH_3_ deposition around an intensive poultry farm. They found that the deposition rate decreased from 42 to 5 kg N ha^−1^ yr^−1^ as the distance of the monitoring sites from the farm increased from 15 m to 270 m. They also found that the NH_3_ adsorbed by woodlands within 270 m and 1 km from the source accounted for 3.2% and 10% of the annual emissions from the farm, respectively. Walker *et al*.^14^ found that NH_3_ dry deposition rates increased from 16 kg N ha^−1^ yr^−1^ at 500 m to 145 kg N ha^−1^ yr^−1^ at 10 m from a commercial swine production facility. The accumulated NH_3_ dry deposition over the nearest 500 m from the barn/lagoon complex accounted for 10.4% of annual emissions^14^. Hao *et al*.^15^ measured NH_3_ deposition downwind of a large cattle feedlot in Canada and found that NH_3_ deposition ranged from 104 kg N ha^−1^ yr^−1^ at the feedlot boundary to 49 kg N ha^−1^ yr^−1^ 700 m from the source.

Cattle feedlots are large NH_3_ hotspots in Australia^16^,^17^ with annual emissions of approximately 33,200 tonne NH_3_-N based on an emission rate of 104 g NH_3_-N head^−1^ d^−1^ and 875,000 beef cattle in Australian feedlots^18^. However, little is known regarding the local dry deposition of NH_3_ surrounding these hotspots. We conducted a field study with the aim of quantifying NH_3_ dry deposition within 1 km of the edge of a commercial feedlot in Victoria, Australia from April to June 2015. We measured NH_3_ concentrations at five locations along a transect downwind within 1 km of the feedlot and calculated NH_3_ deposition fluxes using a well tested bi-directional NH_3_ exchange model with empirical parameters.

## Results

**Figure 1:**
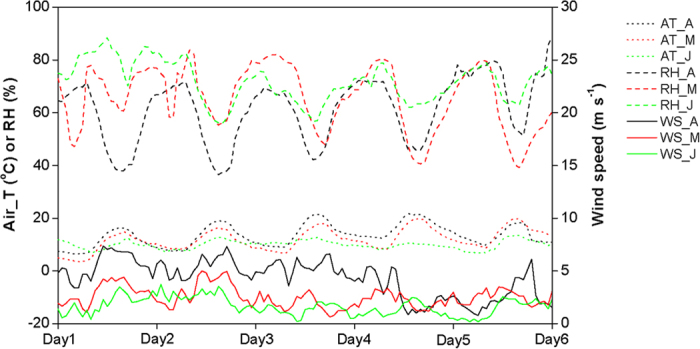
Air temperature (AT), relative humidity (RH) and wind speed (WS) during the sampling periods in April (A), May (M) and June (J).

The dynamics of hourly air temperature, relative humidity and wind speed during the three sampling periods in April, May and June are shown in Fig. 1. The mean daily maximum/minimum temperatures were 21.9/6.4 °C, 19.8/3.5 °C, 13.5/5.3 °C respectively, during the sampling periods of April, May and June, which were 4 °C, on average, lower than the corresponding annual means of the daily maximum/minimum temperatures for the year 2015^19^. The mean wind speeds during the three sampling periods were 4.3, 2.6 and 1.9 m s^−1^ respectively. The averaged wind speed during the three periods (3.0 m s^−1^) was comparable to the annual mean wind speed in 2015 (3.0 m s^−1^)^19^. The mean values of relative humidity during the three periods were 61.1, 81.1 and 71.8% respectively, with a mean of 71%, which was approximately one-third higher than the annual mean in 2015^19^.

**Figure 2:**
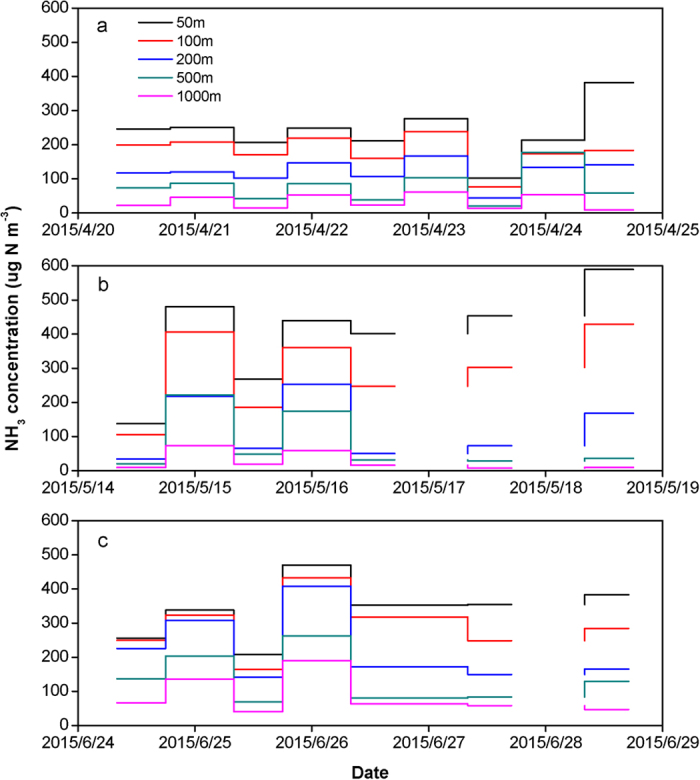
NH_3_ concentrations in April (**a**), May (**b**) and June (**c**) at five downwind sites within 1 km from the feedlot.

**Figure 3:**
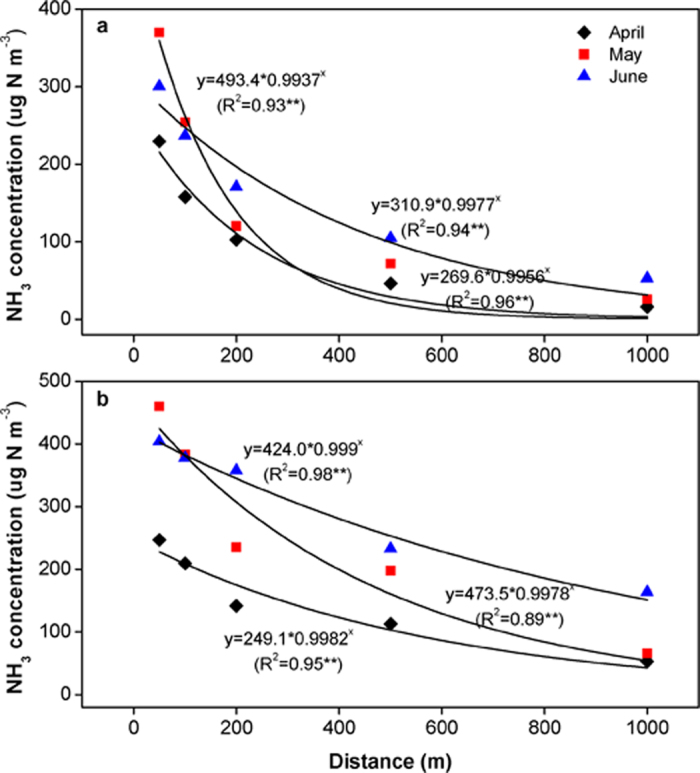
Relationships between the daytime (**a**) and the nighttime (**b**) NH_3_ concentrations and distances from the feedlot (**Significant at 0.01 level).

The measured NH_3_ concentrations showed large spatial and temporal variation (Fig. 2). During the three sampling periods, the mean daytime and nighttime (daytime/nighttime) NH_3_ concentrations were 300/370, 217/324, 117/245, 61.5/181 and 27.2/94.2 μg N m^−3^ at the distance of 50, 100, 200, 500 and 1000 m downwind from the feedlot, respectively. From 50 m downwind to 1 km downwind, NH_3_ concentrations decreased by 74 to 97% during the daytime, and 60 to 87% during the nighttime. NH_3_ concentrations decreased exponentially with distance away from the feedlot (Fig. 3). This indicates, during the period of transporting NH_3_ from the feedlot to the sampling sites, that NH_3_ deposition and NH_3_ dispersion might occur^20^. NH_3_ concentrations were observed the highest at night under stable atmospheric conditions when low dispersion of the NH_3_ plume from the feedlot occurred. The average NH_3_ concentrations during the nighttime were 1.1–6.0 times of those during the daytime. The ratio of NH_3_ concentration during the nighttime to that during the daytime increased with the distance away from the feedlot, due to the faster decrease of NH_3_ concentration with distance away from the feedlot during the daytime than during the nighttime (Fig. 3). This suggests that sampling NH_3_ concentrations separately during the daytime and nighttime is required.

**Figure 4:**
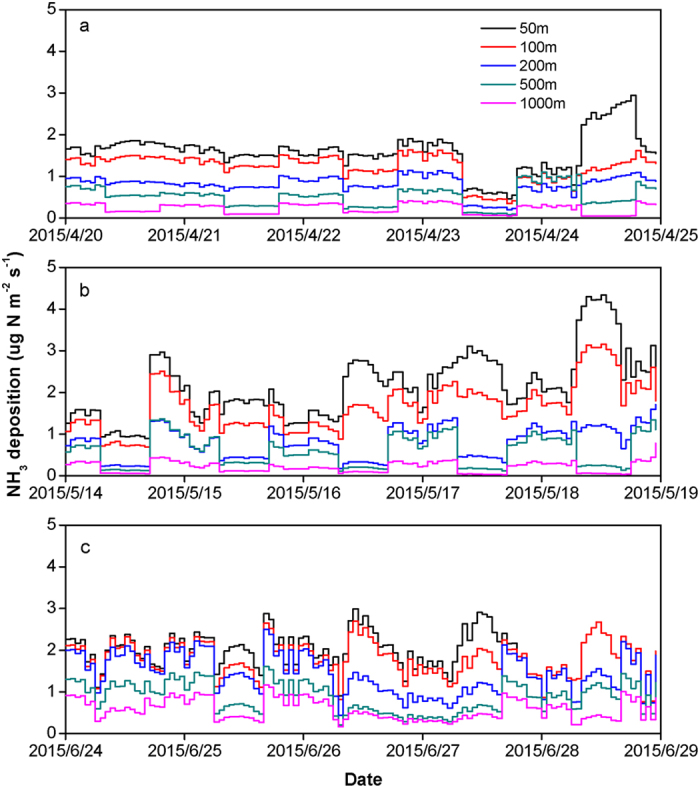
Modelled hourly NH_3_ dry deposition fluxes during the three sampling periods in April (**a**), May (**b**) and June (**c**) for the cropland.

The NH_3_ deposition fluxes also showed large spatial and temporal variations (Fig. 4), ranging from 0.05 to 2.94, 0.03 to 4.34 and 0.03 to 4.34 μg N m^−2^ s^−1^ in April, May and June respectively for the cropland. The NH_3_ deposition fluxes decreased with the distance away from the feedlot. Higher NH_3_ deposition fluxes were generally found during the daytime than during the nighttime at the 50 m site, but this trend was reversed at the 200 m to 1000 m sites. This may be because that the difference of NH_3_ concentration between daytime and nighttime was relatively small at the 50 m site (Fig. 4) and therefore higher wind speed during the daytime favored lower aerodynamic resistance (*R*_*a*_) and caused higher deposition flux in the daytime^14^. But the much higher NH_3_ concentration during the nighttime than during the daytime at 200 m, 500 m and 1000 m sites made NH_3_ concentration the major factor affecting NH_3_ deposition flux.

The mean NH_3_ fluxes in the three sampling periods in April, May and June under the land use types of cropland, grassland and pasture are summarized in Table 1. Similar to NH_3_ concentration, the mean NH_3_ flux also decreased exponentially with the distance away from the feedlot. The variation of the mean NH_3_ deposition fluxes among the three land use types was small. The similarity may be ascribed to the small differences of input parameters (e.g. *R*_*a*_, quasi laminar boundary layer resistance (*R*_*b*_), stomatal resistance (*R*_*s*_), cuticular resistance (*R*_*w*_)) that were used for calculating NH_3_ bi-directional exchanges for the three land use types^21^,^22^,^23^ (see the Supplementary Information for more details).

We estimated the annual NH_3_ dry deposition rates at the downwind sites by assuming that the mean NH_3_ deposition fluxes during April - June for cropland, grassland and pasture represented the annual average of NH_3_ deposition fluxes. The estimated annual NH_3_ deposition rates were 614, 496, 322, 210 and 106 kg N ha^−1^ yr^−1^ at the downwind sites with 50, 100, 200, 500 and 1000 m respectively, from the feedlot, provided that wind direction was constant. In fact, NH_3_ deposition mostly occurred in the downwind areas of the feedlot in this study since the NH_3_ concentration was very low or could not be detected in the upwind direction. Due to the frequent changes in wind direction in the studied region, we calculated the annual NH_3_ deposition in the downwind areas of eight major wind directions (Table 2) by integrated the site-specific NH_3_ dry deposition rates in the downwind transect. By summation, the estimated total NH_3_-N deposition in the areas within 1 km from the feedlot was 53.9 tonne yr^−1^, or 67.5 kg N ha^−1^ yr^−1^ as an area-weighted mean.

## Discussion

The estimated total annual NH_3_ deposition in the area within 1 km away from the studied feedlot accounted for 8.1% of the annual NH_3_ emissions from the feedlot (664 tonne NH_3_-N yr^−1^). This was comparable to that reported by Fowler *et al*.^13^, who estimated that 5–10% of NH_3_ emissions were dry deposited within 1 km of a poultry farm. However, some other studies reported higher fractions of emissions deposited locally. For example, Hao *et al*.^24^ estimated that 16% of the total NH_3_ emitted from a 25,000-head cattle feedlot was deposited to the soil within 1 km of the feedlot. Similarly, Walker *et al*.^14^ calculated that 10.4% of the emitted NH_3_ was dry deposited within 500 m of the emission source. Modelling results have shown that the fraction of local deposited NH_3_ emissions ranged from 2% to 55% within 1 km of the source, which mainly depending on source height, wind speed, atmospheric stability, structure of the surrounding canopies and surface resistance^20^, though most estimates are generally smaller than 20%^1^,^2^. One possible reason for the smaller fraction of emissions deposited locally in this study could be attributed to the relatively high wind speed at our site (with an annual mean of 3 m s^−1^ at 2 m height). High wind speed usually favors a high NH_3_ emission rate^18^, but may also cause fast dispersion and dilution of the NH_3_ plume and thus cause low NH_3_ concentration as well as low NH_3_ dry deposition in the downwind areas^20^. The reduced NH_3_ deposition in the downwind areas and the increased NH_3_ emission rate from feedlot due to high wind speed then may have resulted in a relatively low fraction of locally deposited emissions.

For the remaining 92% of the NH_3_ emitted from the studied feedlot, one possible fate may be that it was transported to the mixing layer in the downwind regions of the feedlot by turbulent dispersion and advection. The depth of the mixing layer has been reported to range from 100 m to up to 1500 m^25^,^26^. Usually, the larger the depth of the mixing layer, the more favourable the mixing of air pollutants with elevation^25^,^26^. In May of 2015 an investigation of NH_3_ concentration around the same feedlot as this study, using the airborne technology, found NH_3_ concentration to range from 470 ppb (294 μg N m^−3^) at 0.5 km downwind of the feedlot to 25 ppb (16 μg N m^−3^) at 6.5 km downwind of the feedlot at 35 m above ground, and as high as 40 ppb (25 μg N m^−3^) at 310 m above the ground along the boundary of the feedlot^27^. These results indicate that most of the emitted NH_3_ may be transported to the mixing layer and could be transported at least 6.5 km from the feedlot. Due to the small emission intensities of acidic gases (e.g., nitric oxides, sulphur dioxide) from industrial and transport sources and nearly no aerosol pollution in the region of the feedlot, the transformation of NH_3_ to particulate NH_4_^+^ could be ignored in the neighbourhood of the feedlot. Therefore, it is also worthwhile to further investigate NH_3_ deposition and related environmental effects in the downwind areas 6.5 km or more from intensive feedlots.

The measured NH_3_ concentrations and estimated NH_3_ deposition rates 1 km from the cattle feedlot in this study were higher than those reported from poultry farm or swine production facilities^13^,^14^. For example, the annual mean NH_3_ concentration was 19 to 52 μg N m^−3^ at a distance of 15 m from a poultry farm (emission intensity: 4.8 tonne NH_3_-N yr^−1^) in the UK and declined to background concentrations of 0.8 to 1.6 μg N m^−3^ at a distance of 270 m, while NH_3_ deposition decreased from 42 kg N ha^−1^ yr^−1^ at 15 m to 5 kg N ha^−1^ yr^−1^ at 270 m with an average of 7 kg N ha^−1^ yr^−1^ in the area within 300 m of the poultry farm^13^. Furthermore, the measured NH_3_ concentration ranged from 139 μg N m^−3^ at a distance of 10 m from a swine production facility in eastern North Carolina (emission intensity: 28.2 tonne NH_3_-N yr^−1^) to 10.7 μg N m^−3^ at 698 m, while the mean NH_3_ deposition ranged from 26 to 52 kg N ha^−1^ yr^−1^ within 500 m from the source^14^. As the estimated NH_3_ emission intensity of the source in this study (664 tonne NH_3_-N yr^−1^ based on 17,500 head of cattle and an emission factor of 104 g NH_3_-N head^−1^ d^−1^) was also higher than those in the above two studies, it can be concluded that the sources with high NH_3_ emission intensities will lead to high NH_3_ deposition in the surrounding area. This conclusion is also supported by Hao *et al*.^24^, who reported much higher NH_3_ deposition around a 25,000-head beef feedlot (emission intensity: 235 tonne NH_3_-N yr^−1^) in Alberta, Canada, which ranged from 120 kg N ha^−1^ yr^−1^ 50 m from the source to 20.8 kg N ha^−1^ yr^−1^ 1 km from the feedlot.

It should be noted that the study we report here is a pilot study to apply the bi-directional NH_3_ exchange model to estimate NH_3_ deposition near a feedlot in Australia. Our calculated NH_3_ deposition is still subject to uncertainty in the model input parameters (*R*_*a*_, *R*_*b*_, *R*_*s*_, *R*_*w*_, *R*_*g*_, *χ*_*s*_ and *χ*_*g*_, see the Supplementary Information for the definitions of these parameters) due to that parameterization of these variables in our study were mainly using the equations or empirical values based American or European researches. For evaluation of the whole model, we also calculated NH_3_ dry deposition velocity by dividing the NH_3_ deposition flux by NH_3_ concentration since no NH_3_ emission flux occurred in this study. The NH_3_ deposition velocities were on average 0.5–0.6 cm s^−1^ for cropland, pasture and grassland around the feedlot. These deposition velocities are comparable with those published mean NH_3_ deposition velocities for cropland (0.4–0.8 cm s^−1^)^28^,^29^, pasture (~0.8 cm s^−1^)^30^ and grassland surfaces (0.5–1.0 cm s^−1^)^28^. Therefore, though there are some uncertainties, the calculated NH_3_ deposition fluxes in this study are still in a reasonable range. Due to the difficulty in accessing the cropland to install the NH_3_ samplers in the growing season, we only conducted NH_3 _deposition sampling during three months. Underestimation may exist when using these three months data to estimate NH_3_ deposition for the whole year. Firstly, due to that the mean air temperature during the three sampling periods in the study was 4 °C lower than the annual mean of air temperature and NH_3_ emission rate is positively correlated with air temperature^18^, the NH_3_ emission intensity and thus the measured NH_3_ concentration during the sampling periods may be lower than the annual means, which means the annual NH_3_ deposition rate at the sampling sites might be underestimated. Secondly, during the three months of sampling periods, only one month was classified into growing season and the other two months classified into un-growing season. In fact, there are half growing season and half un-growing season in a year in the studied region. Because the *R*_*s*_ and *R*_*w*_ are smaller in growing season than those in un-growing season, which favors faster deposition of NH_3_ in growing season than those in un-growing season, the reduced duration of growing season (by 17%) in a year may cause another underestimate of the annual NH_3_ deposition rates.

Our study and previous investigations all indicated that there was high NH_3_ deposition around intensive feedlots or animal farms, which was ranged from 20 to 120 kg N ha^−1^ yr^−1^ and usually higher than the critical loads (10–15 kg N ha^−1^ yr^−1^) of N deposition for most natural or semi-natural ecosystems^31^. Former studies had documented that loss of biodiversity, soil acidification, increase of soil N_2_O emissions could occur in natural and semi-natural ecosystems with increased atmospheric N deposition^6^,^7^,^8^,^32^. Therefore, considering the high NH_3_ deposition around the feedlots or animal facilities, comprehensive studies should be conducted to evaluate the impacts of NH_3_ deposition on the surrounding natural or semi-natural ecosystems, especially in those regions already suffering from high background N deposition (e.g. Eastern China^33^, Western Europe^28^). For the croplands or pastures around the feedlots or animal facilities, NH_3_ deposition is an important N source and researches also need be conducted to reduce N fertilizer application rate by considering N input from NH_3_ deposition in fertilizer recommendation so as to avoid excessive N fertilizer application.

## Methods

**Figure 5:**
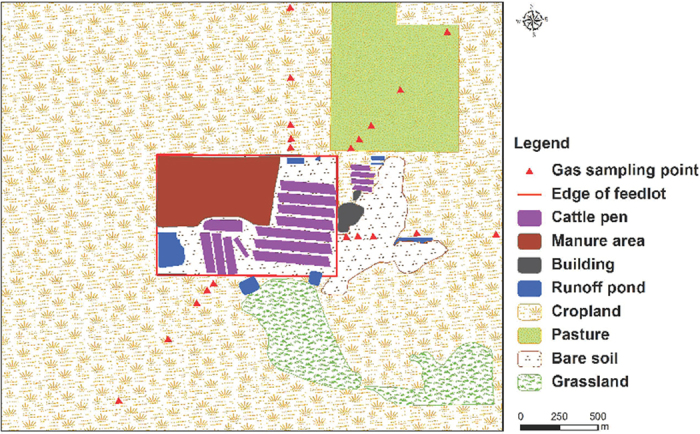
Land use types and locations of NH_3_ samplers within 1 km of the feedlot. Map was drawn using ArcGIS (version 10.0, http://www.arcgis.com).

The experiment was conducted at a typical intensive cattle feedlot, in northeastern Victoria, Australia (Fig. 5). The region has a Mediterranean climate, with long hot summers and mild wet winters^19^. From 2004 to 2015 the mean maximum/minimum temperature was 23.1/9.0 °C and mean annual precipitation was 355 mm^19^. The feedlot area was approximately 93 ha (1,230 m in the east-west direction and 760 m in the north-south direction) including cattle pens, manure stockpiles, bare soil or roads and effluent ponds. The feedlot held approximately 17,500 cattle during the study period. The cattle were 1–1.5 yr of age, European breeds, mostly Angus and Angus cross, with an average body weight of 396 ± 5.3 kg. The cattle consumed an average of 10.2 kg dry matter daily of a finishing ration of barley (*Hordeum vulgare* L.) and grass hay^18^. The area surrounding the feedlot was mainly cropland planted to wheat with sheep pasture lying to the northeast and grassland to the south (Fig. 5). The wheat croplands around the feedlot were usually fertilized (urea was used as basal nitrogen fertilizer at an application rate of approximately 50 kg ha^−1^) and sown in June and harvested in October or November, and fallowed from November or December until May in the following year. The plants in the pasture and grassland were usually growing during June to September.

The NH_3_ deposition measurement was conducted during three sampling periods in 2015 (20^th^ to 24^th^ of April, 14^th^ to 18^th^ of May and 24^th^ to 29^th^ of June). The NH_3_ concentration was measured by a denuder system for long-term ammonia sampling (DELTA)^34^,^35^,^36^. A low-volume pump (D210, TCS Micropumps Ltd., UK) was used to draw air at a rate of 0.2–0.4 L min^−1^. Prior to the measurement, two denuders, connected in series to adsorb the NH_3_ in the air, were treated with a solution of 5% (m/v) citric acid in methanol. When the air was drawn through the denuder train, NH_3_ gas was adsorbed to the inner surface of the denuders. The total sampled air volume was recorded by a dry gas meter (SK25, Kimmon Manufacturing Co., Ltd., Japan). The gas meter was checked at the start of each sampling period using a gas flow meter to ensure that the recorded gas volumes were correct. The denuders, gas meter and pump were connected in sequence with short (2–3 cm) silicone tubes, and were fixed in a PVC box (40 × 30 × 50 cm) with the inlet of the denuder train exposed to the ambient air. During NH_3_ sampling period, the PVC box was attached to a pole at a height of 1.5 m above the ground. The denuder trains were changed two times each day, based on the day and night cycle. During each sampling period, five daytime (8:00 am to 5:00 pm) and nighttime (6:00 pm to 7:00 am in the following day) NH_3_ samples were collected continuously at each location. The samples were stored at 4 °C and analysed at the end of each sampling period at an off-site laboratory. The NH_3_ denuders were extracted with milliQ^®^ water for 1 h, and the extraction was analysed for NH_4_^+^-N content by segmented flow analyzer (Skalar SAN^++^, Netherlands). The detection limit of the DELTA system, calculated as 2σ (two times of the standard deviation) of the field blanks, was 0.28 μg N m^−3^.

The NH_3_ concentrations were measured at five locations downwind of the feedlot. The sampling locations were selected along a transect downwind of the centre of the feedlot, with a distance of 50, 100, 200, 500 and 1000 m from the fence line of the feedlot (Fig. 5). The sampling locations were determined according to daily predominant wind direction, measured by a three-dimensional (3-D) sonic anemometer, so that downwind NH_3_ concentrations from feedlot were measured. The actual sampling duration for a sampling site was then recorded only when the site was located at downwind of the feedlot. The measured NH_3_ concentrations were discarded if wind direction changed and the downwind sampling duration was less than 50% of the total sampling duration.

A weather station coupled with a 3-D sonic anemometer (CSAT3, Campbell Scientific, Logan, USA) was set up at a height of 3.3 m above the ground located to the east of the feedlot. Fifteen-min averaged air temperature, wind speed, friction velocity, Monin-Obuhkov length and relative humidity were recorded at 10 Hz. The raw data was processed to hourly average data using SAS software (SAS 9.4, SAS Institute Inc., Cary, NC, USA).

A well tested bi-directional NH_3_ exchange model, which is called the two-layer canopy compensation point model^23^,^37^, was used to estimate NH_3_ dry deposition around the feedlot. Similar to most of the dry deposition models, the bi-directional flux model is based on a formula analogous to Ohm’s law in electrical circuits where flux (analogous to current) is calculated by dividing the concentration difference (analogous to voltage) by the deposition resistance (analogous to electrical resistance)^21^,^38^. According to Nemitz *et al*.^37^, the total NH_3_ flux (*F*_*t*_) is the sum of bi-directional exchange with the leaf stomata (*F*_*s*_), deposition to the leaf cuticle (*F*_*w*_) and bi-directional exchange with ground (*F*_*g*_). Among these pathways, *F*_*s*_ and *F*_*w*_ occur parallelly in the canopy layer and can be summed as the canopy flux (*F*_*f*_), while *F*_*g*_ occurs in the ground layer^37^. The relations and definition of each of the fluxes are shown in the following equations^37^:


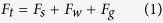



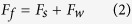



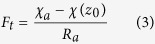



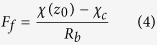



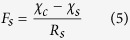



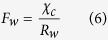



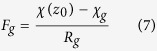


where 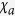 is the measured NH_3_ concentration at the height of 1.5 m above the ground level, 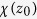 is the NH_3_ concentration at height of *d* + *z*_*0*_, *d* is the zero-plane displacement height, *z*_*0*_ is the surface roughness length, *R*_*a*_ is the aerodynamic resistance, a function of the vertical turbulent diffusive transport through the atmosphere, 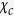 the canopy NH_3_ compensation point, *R*_*b*_ the quasi laminar boundary layer resistance, 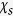 the stomatal compensation point, *R*_*s*_ the stomatal resistance, *R*_*w*_ the cuticular resistance, 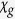 the ground layer NH_3_ compensation point and *R*_*g*_ the in-canopy resistance to the ground. Based on the equations of (1), (3), (4) and (7), 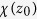 can be calculated using the following equation:


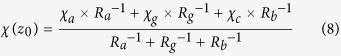


Based on the equations of (2), (4), (5), (6) and (8), the equation of 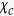 can be deduced as the following:


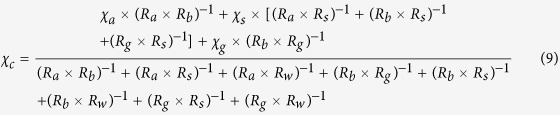


The parameters of *R*_*a*_, *R*_*b*_, *R*_*g*_, *R*_*s*_, *R*_*w*_, 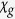 and 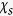 were calculated according to Wesely^21^, Erisman & Draaijers^22^ and Massad *et al*.^23^ (see the Supplementary Information for more details). Therefore, according to equations (3,8 and 9), the total NH_3_ dry deposition flux was calculated. Hourly NH_3_ dry deposition flux was calculated based on the hourly meteorological data. As we did not measure hourly NH_3_ concentrations, their values in a sampling event were assumed to be equal to the corresponding daytime or nighttime NH_3_ concentration measured by the DELTA system. The missing data of NH_3_ concentrations in a sampling event were interpolated with the mean daytime or nighttime NH_3_ concentrations measured during the same sampling period. The R software v3.13^39^ was used for regression analyses. The significance level was set as p < 0.05.

**Figure 6:**
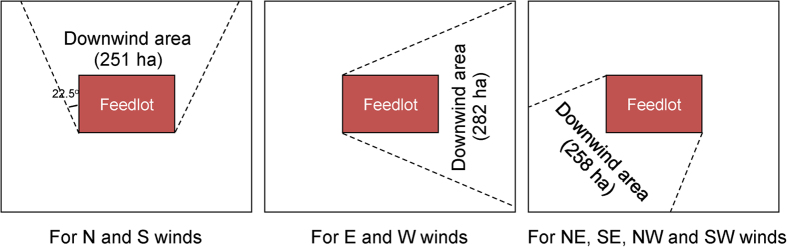
Downwind areas within 1 km of the feedlot for different wind directions (The wind directions for north (N), northeast (NE), east (E), southeast (SE), south (S), southwest (SW), west (W), northwest (NW) are −22.5° to 22.5°, 22.5° to 67.5°, 67.5° to 112.5°, 112.5° to 157.5°, 157.5° to 202.5°, 202.5° to 247.5°, 247.5° to 292.5° and 292.5° to 337.5° respectively).

The following steps were used to integrate the site-specific NH_3_ dry deposition rates in the downwind transect to the total NH_3_ deposition in the downwind area within 1 km from the feedlot. Firstly, using the correlation between the measured NH_3_ deposition rate and distance from the feedlot, we estimated the deposition rates at 200, 300, 400, 600, 700, 800 and 900 m from the feedlot. Secondly, due to that NH_3_ deposition occurred mostly in the downwind direction as the NH_3_ concentration was very low or could not be detected, we divided the total area within 1 km from the feedlot into eight downwind areas based on the eight major wind directions. Here a downwind area is defined as the area within 1 km from the feedlot that can be affected by the feedlot NH_3_ plumes transported by a certain direction of wind. Examples for dividing downwind areas are shown in Fig. 6. We further divided each downwind area into 11 sub-areas, that is 1) area within 50 m from the feedlot, 2) area within 50 to 100 m from the feedlot, 3) area within 100 to 200 m from the feedlot, 4) area within 200 to 300 m from the feedlot, 5) area within 300 to 400 m from the feedlot, 6) area within 400 to 500 m from the feedlot, 7) area within 500 to 600 m from the feedlot, 8) area within 600 to 700 m from the feedlot, 9) area within 700 to 800 m from the feedlot, 10) area within 800 to 900 m from the feedlot, and 11) area within 900 to 1000 m from the feedlot. For the sub-area 1), we assumed that the NH_3_ deposition rate in this area was equal to the NH_3_ deposition rate at 50 m from the feedlot. NH_3_ deposition rates in other sub-areas were assumed to be equal to the mean of the deposition rates at the nearest and longest distances from the feedlot in the specified sub-area. For example, for sub-area 2), the NH_3_ deposition rate in the area was assumed to be equal to the mean of the deposition rates at 50 m and 100 m from the feedlot. The NH_3_ deposition rate in each sub-area then can be calculated by multiplying the site-specific NH_3_ deposition rate with the size of each sub-area. Thirdly, we calculated NH_3_ deposition in a downwind area by multiplying the frequency of a wind direction in a year with the summed NH_3_ deposition in 11 sub-areas of this downwind area. By summation, we can get the total NH_3_ deposition within 1 km from the feedlot using the following equation:


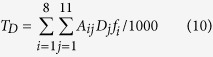


where *T*_*D*_ is the total NH_3_ deposition (tonne N yr^−1^) in the area within 1 km from the feedlot, *A*_*ij*_ is the size (ha) of the *j*^*th*^ sub-area of the *i*^*th*^ downwind area, *D*_*j*_ the NH_3_ deposition rate (kg N ha^−1^ yr^−1^) in the *j*^*th*^ sub-area, *f*_*i*_ is the frequency of the *i*^*th*^ wind direction in a year and and 1000 the unit conversion factor. The area-weighted NH_3_ deposition rate is then calculated by divided the total NH_3_ dry deposition around the feedlot by the total area within 1 km from the feedlot.

## Additional Information

**How to cite this article**: Shen, J. *et al*. Ammonia deposition in the neighbourhood of an intensive cattle feedlot in Victoria, Australia. *Sci. Rep.* **6**, 32793; doi: 10.1038/srep32793 (2016).

## Supplementary Material

Supplementary Information

## Figures and Tables

**Table 1:** Mean NH_3_ deposition fluxes (±standard deviation, μg N m^−2^ s^−1^) under different land use types during the sampling periods in April, May and June for the five sampling sites.

Site	April			May			June		
	Cropland	Grassland	Pasture	Cropland	Grassland	Pasture	Cropland	Grassland	Pasture
50 m	1.58 ± 0.47	1.71 ± 0.54	1.70 ± 0.54	2.15 ± 0.81	2.38 ± 1.04	2.37 ± 1.04	2.07 ± 0.51	2.00 ± 0.45	2.00 ± 0.45
100 m	1.23 ± 0.31	1.32 ± 0.34	1.31 ± 0.34	1.65 ± 0.59	1.82 ± 0.72	1.80 ± 0.72	1.79 ± 0.43	1.76 ± 0.38	1.75 ± 0.38
200 m	0.81 ± 0.21	0.87 ± 0.22	0.87 ± 0.22	0.80 ± 0.37	0.87 ± 0.39	0.86 ± 0.39	1.36 ± 0.46	1.41 ± 0.46	1.40 ± 0.46
500 m	0.52 ± 0.24	0.56 ± 0.23	0.55 ± 0.23	0.58 ± 0.37	0.62 ± 0.37	0.61 ± 0.37	0.83 ± 0.36	0.87 ± 0.35	0.86 ± 0.35
1 km	0.23 ± 0.12	0.25 ± 0.11	0.24 ± 0.11	0.20 ± 0.13	0.22 ± 0.12	0.21 ± 0.12	0.55 ± 0.25	0.57 ± 0.25	0.56 ± 0.25

**Table 2:** Annual NH_3_ deposition in the eight major downwind areas within 1 km from the feedlot.

Wind direction	Frequency (%)	Downwind area (ha)	NH_3_ deposition (tonne N yr^−1^)
North	8†	251	4.8
South	19	251	11.3
East	6	282	4.3
West	13	282	9.3
Northeast	13	258	7.0
Northwest	9	258	4.8
Southeast	8	258	4.3
Southwest	15	258	8.1
Total	91	2097	53.9

^†^Data from Bureau of Meteorology of Australia as an average of 2010 to 2014.
