# Supplementary material for: Ammonia deposition in the neighbourhood of an intensive cattle feedlot in Victoria, Australia
Source: Sci Rep. 2016 Sep 7;6:32793. doi: 10.1038/srep32793 (PMC5013440; doi:10.1038/srep32793)
Supplement: Supplementary Information [file srep32793-s1.doc]

**Supplementary Information for**

**Ammonia deposition in the neighbourhood of an intensive cattle feedlot in Victoria, Australia**

Jianlin Shen^1, 2, *^, Deli Chen^2, *^, Mei Bai^2^, Jianlei Sun^2^, Trevor Coates^2^, Shu Kee Lam^2^, Yong Li^1^

^1^ Key Laboratory of Agro-Ecological Processes in Subtropical Regions, Institute of Subtropical Agriculture, Chinese Academy of Sciences, Changsha 410125, China

^2^ Faculty of Veterinary and Agricultural Sciences, The University of Melbourne, Victoria 3010, Australia.

* Correspondence to: Dr. Jianlin Shen

Institute of Subtropical Agriculture, Chinese Academy of Sciences, Hunan 410125, China

Tel: +86-731-84619722; Fax: +86-731-84612685; E-mail: jlshen@isa.ac.cn

* Or correspondence to: Prof. Deli Chen

Faculty of Veterinary and Agricultural Sciences, The University of Melbourne, Victoria 3010, Australia

Tel: +61 3 8344 8148; Fax: +61 3 8344 8148; E-mail: delichen@unimelb.edu.au

1. Parameterization of the deposition resistances (*R_a_*, *R_b_*, *R_g_*, *R_s_* and *R_w_*)

1. 1 Aerodynamic resistance (*R_a_*)

The parameterization of *R_a_* was according to Erisman & Draaijers^1^ using the following equation:

$R_{a}(z)=(ku_{*})^{-1}[\ln(\frac{z-d}{z_{0}})-\psi_{h}(\frac{z-d}{L})+\psi_{h}(\frac{z_{0}}{L})]$ (1)

where *z* is the measuring sensor height, *k* the von Karman constant (0.41), *u** the friction velocity, *d* the zero-plane displacement height, *z_0_* the roughness length, *ψ_h_* the integrated stability functions for entrained scalars and L the Monin-Obukhov length. In this study, *u** and *L* were calculated based on data from the three dimensional sonic anemometer located in the studied feedlot. *d* and *z_0_* were set to be 0 m and 0.01 m respectively for the bare soil and 0.67 and 0.1 times of the plant height respectively when the soils were coved with plants. *ψ_h_* was parameterized using the following equation:

For stable condition,

$\psi_{h}(\frac{z-d}{L})=-5.2(z-d)/L$ (2)

For unstable condition,

$\psi_{h}(\frac{z-d}{L})=2\ln((1+x^{2})/2)$ (3)

where

$x=[1-16(z-d)/L]^{0.25}$ (4)

1.2 Quasi laminar boundary layer resistance (*R_b_*)

The *R_b_* was also parameterized based on easily measurable meteorological parameters according to Erisman & Draaijers^1^. The equation for *R_b_* is as follows:

$R_{b}=(2/ku_{^{*}})(S_{c}/\text{Pr})^{2/3}$ (5)

where *k* is the von Karman constant, *u** the friction velocity, Pr the Prandtl number (0.72), *S_c_* the Schimidt number, which is calculated according to the following equation:

$S_{c}=\nu/D_{NH3}$ (6)

where *ν* is the kinematic viscosity of air (~ 1.56 × 10^-5^ m^2^ s^-1^ at 25 ^o^C), *D_NH3_* is the molecular diffusivity of NH_3_ in air (~2.32 × 10^-5^ m^2^ s^-1^ at 25 ^o^C) ^2^.

1.3 In-canopy resistance to the ground (*R_g_*)

According to Massad et al.^3^, the *R_g_* is the sum of the aerodynamic resistance within the canopy (*R_ac_*) and the soil boundary layer resistance (*R_bg_*). In this study, *R_ac_* was set as 10 s m^-1^ for the cropland, grassland and pasture in April and May (non-growing season) and June (growing season, but with short or sparse plants) according to Zhang *et al.*^4^, while *R_bg_* was set as 100 s m^-1^ for all the land use types according to Erisman & van Pul^5^.

1.4 Stomatal resistance (*R_s_*)

The parameterization of *R_s_* was according to Wesely^6^ using the following equation:

$R_{s}=r_{i}\left\{1+[200(G+0.1)^{-1}]^{2}\right\}\left\{400[T_{s}(40-T_{s})]^{-1}\right\}$ (7)

where *r_i_* is the specified stomatal resistance for a specified land use type and season according to Wesely^6^, *G* is the solar irradiation in W m^-2^ and *T_s_* is the surface air temperature (^o^C).

1.5 Cuticular resistance (*R_w_*)

*R_w_* was set very high (1×10^25^ s m^-1^) for the cropland in April and May (non-growing season) as the ground was covered with bare soils. In June (growing season) for cropland and in April to June for grassland and pasture, *R_w_* was parameterized using the following equation according to Massad et al.^3^:

$R_{w}=31.5\times(AR)\times e^{a(100-RH)}$ (8)

where *AR* is the mole ratio of total acid (SO_2_, HNO_3_ and HCl) to NH_3_ in the air (set as 0.1 in this study), *a* is land use specified parameter (0.148 for cropland and 0.176 for grassland and pasture) and *RH* is the relative humidity (%).

2 Parameterization of compensation points ($\chi_{g}$ and $\chi_{s}$) for NH_3_ bi-directional flux modelling

2.1 Ground layer compensation point ($\chi_{g}$)

According to Nemitz *et al.*^7^, $\chi_{g}$ was parameterized using the following equation:

$\chi_{g}=\frac{161500}{T_{g}}\exp(-10378T_{g}^{-1})\frac{[NH_{4}^{+}]}{[H^{+}]}$ (9)

Where *T_g_* is the temperature at ground surface (K), [NH_4_^+^] and [H^+^] are the NH_4_^+^ and H^+^ concentrations in the soil solution, respectively. In this study, as no NH_4_^+^ and H^+^ concentrations in the soil solution were measured, the specified ratios of [NH_4_^+^]/[H^+^] under different surface conditions according to Massad *et al.*^3^ were used (Table S1). In this study, due to no measurement for *T_g_*, equal of *T_g_* to air temperature was assumed.

**Table S1.** Ratios of [NH_4_^+^] to [H^+^] for parameterization of under different land use types

| Month | Land use | Management | Vegetation | [NH_4_^+^]/[H^+^] |
| --- | --- | --- | --- | --- |
| April | Cropland | Background | None | 500 |
|  | Grassland | Un-managed | Grass | N/A |
|  | Pasture | Background | None | 500 |
| May | Cropland | Background | None | 500 |
|  | Grassland | Un-managed | Grass | N/A |
|  | Pasture | Background | None | 500 |
| June | Cropland | Fertilized | Wheat | (Napp/(θ_s_*M_N_*l_s_*h_m_)/10^-pH^)*e^(-t/T)^, T=2.88 days^†^ |
|  | Grassland | Un-managed | Grass | N/A |
|  | Pasture | Background | Grass | N/A |

^†^ Napp: Nitrogen fertilization application rate (~25 kg N ha^-1^ in the form of urea in the studied area), θ_s_: soil percentage water content, M_N_: molar mass of nitrogen (14 g mol^-1^), ls: soil layer where fertilizer is applied, h_m_: for converting hectare to m^2^ (=10,000 m^2^), pH: pH of the soil solution after fertilizer application, t: days after fertilizer application. If (Napp/(θ_s_*M_N_*l_s_*h_m_)/10^-pH^)*e^(-t/T)^ is less than 500, then the ratio of [NH_4_^+^] to [H^+^] is assumed to be equal to 500.

2.2 Stomatal compensation point ($\chi_{s}$)

The equation for parameterization of $\chi_{s}$ is similar to that of $\chi_{g}$and is listed as following:

$\chi_{s}=\frac{161500}{T_{l}}\exp(-10378T_{l}^{-1})\frac{[NH_{4}^{+}]}{[H^{+}]}$ (10)

where *T_l_* is leaf temperature (K), [NH_4_^+^] and [H^+^] are the NH_4_^+^ and H^+^ concentrations in leaf apoplast, respectively. In this study, due to no measurement for *T_l_*, equal of *T_l_* to air temperature was assumed. Also, as no measurement of [NH_4_^+^] and [H^+^] in this study, the specified ratios of [NH_4_^+^] to [H^+^] under different surface conditions according to Massad *et al.*^3^ were used (Table S2).

**Table S2.** Ratios of [NH_4_^+^] to [H^+^] for parameterization of $\chi_{s}$ under different land use types

| Month | Land use | Management | Vegetation | [NH_4_^+^]/[H^+^] |
| --- | --- | --- | --- | --- |
| April | Cropland | Background | None | N/A |
|  | Grassland | Un-managed | Grass | 200^†^ |
|  | Pasture | Background | None | N/A |
| May | Cropland | Background | None | N/A |
|  | Grassland | Un-managed | Grass | 200 |
|  | Pasture | Background | None | N/A |
| June | Cropland | Fertilized | Wheat, oil rape | (12.3*Napp+20.3)×e^(-t/T)^, T=2.88 days‡ |
|  | Grassland | Un-managed | Grass | 200 |
|  | Pasture | Background | Grass | 200 |

^†^ Mean values for the published ratios of [NH_4_^+^] to [H^+^] for plants in the semi-natural ecosystems.

‡ Napp: Nitrogen fertilization application rate (~25 kg N ha^-1^ in the form of urea in the studied area), t: days after fertilizer application.

**References**

1. Erisman, J.W. & Draaijers, G.P.J. Atmospheric deposition in relation to acidification and eutrophication in *Studies in Environmental Research* (ed. Draaijers, G.P.J.) Vo. 63, 1–404 (Elsevier, 1995).

2. Massman, W. J. A review of the molecular diffusivities of H_2_O, CO_2_, CH_4_, CO, O_3_, SO_2_, NH_3_, N_2_O, NO, and NO_2_ in air, O_2_ and N_2_ near STP. *Atmos Environ* **32**, 1111–1127 (1998).

3. Massad, R.-S., Nemitz, E. & Sutton, M.A. Review and parameterisation of bi-directional ammonia exchange between vegetation and the atmosphere. *Atmos Chem Phys* **10**, 10359–10386 (2010).

4. Zhang, L., Brook, J.R. & Vet, R. A revised parameterization for gaseous dry deposition in air-quality models. *Atmos Chem Phys* **3**, 2067–2082 (2003).

5. Erisman, J.W. & van Pul, W.A.J. Parameterization of surface resistance for the quantification of atmospheric deposition of acidifying pollutants and ozone. *Atmos Environ* **16**, 2595–2607 (1994).

6. Wesely, M. L. Parameterization of surface resistances to gaseous dry deposition in regional scale numerical models. *Atmos Environ* **23**, 1293–1304 (1989).

7. Nemitz, E., Milford, C., & Sutton, M. A. A two-layer canopy compensation point model for describing bi-directional biosphere-atmosphere exchange of ammonia. *Q J Roy Meteor Soc* **127**, 815–833 (2001).
